# Clinical and socio-demographic characteristics associated with perinatal depression and human immunodeficiency virus

**DOI:** 10.4102/sajpsychiatry.v32i0.2620

**Published:** 2026-03-25

**Authors:** Johanna H.C. Landman, Yumna Minty-Seth

**Affiliations:** 1 Department of Psychiatry, School of Clinical Medicine, Faculty of Health Sciences, University of the Witwatersrand, Johannesburg, South Africa

**Keywords:** perinatal, depression, HIV, EPDS, maternal mental health, pregnancy, psychiatry

## Abstract

**Background:**

Perinatal depression is highly prevalent in South Africa. Human immunodeficiency virus (HIV), a known risk factor for depression, affects a large proportion of South African women of reproductive age. Few studies have compared depressive symptoms and associated risk factors in HIV-positive and HIV-negative pregnant and postpartum women, despite both conditions being linked to adverse maternal and child outcomes.

**Aim:**

To compare socio-demographic and clinical characteristics of HIV-positive and HIV-negative women in the perinatal period attending a maternal mental health clinic.

**Setting:**

The maternal mental health clinic at Chris Hani Baragwanath Academic Hospital in Soweto, Johannesburg.

**Methods:**

A retrospective record review was conducted on 190 patients (HIV-positive: *n* = 40; HIV-negative: *n* = 150) seen at the clinic between January 2024 and April 2025. Data were extracted from the clinic’s REDCap database and analysed.

**Results:**

The prevalence of significant depressive symptoms (Edinburgh Postnatal Depression Scale [EPDS] score > 13) was 43.2% (*n* = 82), regardless of HIV status. High rates of unplanned pregnancies (78.1%, *n* = 145), substance use in pregnancy (19.5%, *n* = 37), and intimate partner violence (15.1%, *n* = 28) were observed regardless of HIV status. Poor social support was significantly more prevalent among HIV-positive women (*p* = 0.042).

**Conclusion:**

Perinatal depressive symptoms were highly prevalent. Human immunodeficiency virus-positive women were more likely to report poor social support, a key risk factor for depressive symptoms. The findings of this study underscore the need for targeted psychosocial interventions.

**Contribution:**

This study encourages further research to explore risk factors associated with perinatal depression, especially in HIV-positive women.

## Introduction

South Africa has one of the highest rates of postpartum depression globally.^1,2^ The estimated prevalence of postpartum depression in South Africa is 39%, significantly exceeding the global prevalence of 17%.^2^ This elevated burden of disease occurs within a broader context of significant social and structural challenges, including high rates of gender-based violence, unemployment, human immunodeficiency virus (HIV) infection, and limited health care services, all of which negatively affect maternal mental and physical health.^3,4,5,6^ Maternal mental health has a significant impact on maternal attachment and child development.^1,7,8^ Depressive disorders are associated with symptoms of lethargy, anhedonia, and psychomotor slowing, which can impair a mother’s capacity to meet her child’s physical and emotional needs.^1^ There is evidence to suggest that children born to women with postpartum depressive disorder have significant developmental delays, especially in language and social development.^1,8^ Long-term consequences for the child may include conduct-related problems, attention deficit hyperactivity disorders, anxiety symptoms, antisocial personality traits, and poor school performance.^8^ In addition, poor bonding associated with maternal depression often leads to early termination of breastfeeding, potentially compromising a child’s nutritional requirements.^7^

Human immunodeficiency virus infection is a well-established risk factor for perinatal depressive disorders.^9,10^ In South Africa, approximately one-fifth of women of reproductive-age are living with HIV.^11^ Factors such as early sexual debut, sexual relationships with older partners, low level of education, lack of condom use, and gender-based violence contribute to the elevated risk of HIV infection among young females.^3,12^

Antenatal and postnatal depressive disorder have been found to be more prevalent among HIV-positive women, as compared to HIV-negative women.^5,13^ However, studies conducted in South Africa have shown substantial variability in reported prevalence rates of perinatal depression between these groups.^5,14,15^ In addition, there is limited research directly comparing established risk factors for perinatal depressive disorders, such as intimate partner violence, unplanned pregnancies, substance use, and poor social support in HIV-positive and HIV-negative perinatal women.^13^ Human immunodeficiency virus further contributes to adverse perinatal outcomes, including preterm labour and having an infant with low birth weight.^9,10^ Rochat et al.^6^ found that newly diagnosed HIV infection was a significant trigger for suicidal ideation among pregnant women in their second half of pregnancy.

Research focusing on HIV-positive women in the perinatal period can, therefore, assist with prioritising and directing the individualised and appropriate delivery of perinatal mental health care services in South Africa. In a resource-limited country, it is important to direct services to high-risk population groups. Furthermore, it may assist in advocating for the integration and prioritisation of specific healthcare services, such as adherence counselling, substance rehabilitation, social support, and mental health care services within antenatal services.

### Aim and objectives

The aim of the study was to describe the socio-demographic and clinical characteristics of HIV-positive and HIV-negative perinatal patients, and to compare these findings across both groups. The factors researched were Edinburgh Postnatal Depression Scale (EPDS) scores, level of social support, prevalence of substance use in pregnancy, prevalence of intimate partner violence, and prevalence of unplanned pregnancies. Additionally, socio-demographic characteristics including age, highest level of education and relationship status of HIV-positive and HIV-negative perinatal individuals were described and compared.

## Research methods and design

### Study design and setting

This was a retrospective review of clinical patient records from the maternal mental health clinic at Chris Hani Baragwanath Academic Hospital (CHBAH) in Soweto, Gauteng. The clinic provides specialised mental health services to pregnant and recently postpartum patients.

### Study population and sampling strategy

Study participants included all women who attended the maternal mental health clinic at CHBAH between 01 January 2024 and 30 April 2025, who were within the perinatal period (pregnant or up to 6 months post-delivery). Patients were excluded if they had incomplete records on the Research Electronic Data Capture (REDCap) database, specifically with respect to HIV status, gestational age, and initial EPDS scores. The minimum sample size was calculated based on 2024 mid-year population prevalence figures of HIV, estimated at approximately 20% among women aged 15–49 years.^11^ A 95% confidence interval with a 5% margin of error was used, considering an estimated total number of 1000 patients for a 5-year period at the maternal mental health clinic according to records. Furthermore, with 10% non-complete rate, the minimum sample size of this study was calculated to be 220 participants. However, several records were incomplete and had to be excluded from the study, resulting in a total of 190 participants being included in this study.

### Data collection

Data were exported from the clinic’s REDCap database. Socio-demographic information (age, highest level of education, and relationship status) and clinical information (EPDS scores at initial visit to clinic, gestation, HIV status, substance use in pregnancy, level of social support, self-reported intimate partner violence and unplanned pregnancy) were exported from the REDCap database to an Excel spreadsheet. Depressive symptoms during the perinatal period were assessed using the EPDS, a 10-item screening instrument developed to identify depressive symptomatology in pregnant and postpartum women.^16,17^ In a South African validation study,^17^ a cut-off score of 11 or higher was found to optimally identify depressive disorders, with a sensitivity of 80% and a specificity of 76.6%. However, a systematic review^17^ indicated that a more conservative cut-off score of > 13 increased the specificity of detecting major depressive disorder to 95%; this higher cut-off was applied in this study.

Information on social support was based on subjective patient self-reports documented during prior clinical interviews. Support was categorised as good if the patient was recorded as receiving care and support from both her partner and family or friends; fair if support was documented from either her partner or family or friends; and poor if no support from either her partner or family or friends was recorded.

### Data analysis

Data were analysed using STATA version 18.5 SE.^18^ Continuous data, including age, gestation and EPDS scores were found to be non-normally distributed using the Shapiro-Wilk test; therefore, they were represented as median and interquartile length. Subsequently, the Wilcox-Mann-Whitney test was used to determine the association between continuous variables and HIV status. Categorical variables, including HIV status, relationship status, highest level of education, substance use in pregnancy, social support, unplanned pregnancies and intimate partner violence were represented as frequencies and percentages. The Fisher’s exact test was used to determine the association between HIV status and categorical variables. Multiple linear regression analysis was conducted for significant Fisher’s exact *p*-values. Statistical significance was set at *p* < 0.05.

### Ethical considerations

Prior to collecting data, written ethics approval was obtained from the Human Research Ethics Committee (Medical) of the University of the Witwatersrand (reference number: M250474). The study was registered on the National Health Research Database (reference number: GP 202503071). In addition, written permission was obtained from the CEO and the Head of the Psychiatric Department of CHBAH to conduct the study. Patient confidentiality was preserved by removing all identifiable personal information when data were extracted from the REDCap database. All patient information was and still remains protected by a secure password on the REDCap database.

## Results

### Socio-demographic and clinical characteristics of the study population

This retrospective study included 190 patients. The overall socio-demographic and clinical characteristics of the study population are summarised in Table 1. The median age of participants was 28 years. The prevalence of HIV-positive status was 21.1% (*n* = 40).

**TABLE 1:** Socio-demographic and clinical characteristics of the study population.

Variables	*n*	%	Median	IQR
*N*	190	-	-	-
Age (years)	-	-	28	22–34
HIV-positive	40	21.1	-	-
EPDS score	-	-	11	5–18
**Significant depressive symptoms: EPDS score > 13**				
% of total (*n*)	82	43.2	-	-
**Relationship status**				
Ongoing relationship	146	78.1	-	-
**Social support**				
Poor	11	5.9	-	-
Fair	81	43.1	-	-
Good	96	51.1	-	-
**Highest level of education**				
Learners with special education needs (LSEN)	8	4.5	-	-
Primary School	10	5.6	-	-
High School	71	40.1	-	-
Matric	66	37.3	-	-
Higher education	22	12.4	-	-
**Substance use in pregnancy**				
Substance use in pregnancy	37	19.5	-	-
**Unplanned pregnancy**				
Unplanned pregnancy	145	76.7	-	-
**Intimate partner violence**				
Intimate partner violence	28	15.1	-	-

Note: Age and EPDS scores are represented as median and interquartile range. Human immunodeficiency virus status, significant depressive symptoms, relationship status, social support, highest level of education, substance use in pregnancy, unplanned pregnancies and intimate partner violence are presented as frequencies (*n*) and percentages (%).

HIV, human immunodeficiency virus; EPDS, Edinburgh Postnatal Depression Scale; IQR, interquartile range.

**FIGURE 1:**
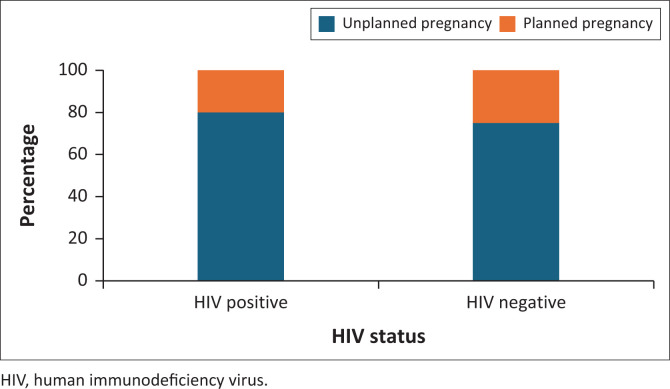
Proportion of human immunodeficiency virus-positive and human immunodeficiency virus-negative patients reporting unplanned and planned pregnancies at Chris Hani Baragwanath maternal mental health clinic.

The median EPDS score was 11, with 43.2% (*n* = 82) of patients having significant depressive symptoms (EPDS score > 13). The majority of patients (78.1%, *n* = 146) reported being in an ongoing relationship with an intimate partner. Poor social support was reported by 5.9% of patients (*n* = 11). Around half of patients indicated having good social support (51.1%, *n* = 96). In addition, around half of patients (49.7%, *n* = 88) had achieved matric-level education (equivalent to the final year of secondary school) or higher. The prevalence of substance use in pregnancy was 19.5% (*n* = 37). A high rate of unplanned pregnancies was reported (76.7%, *n* = 145), as illustrated in Figure 1. Exposure to intimate partner violence was reported by 15.1% (*n* = 28) of participants.

### Comparison of human immunodeficiency virus-negative and human immunodeficiency virus-positive perinatal patients

The socio-demographic and clinical characteristics of HIV-negative and HIV-positive perinatal patients are summarised in Table 2. The median age of HIV-positive patients (32 years) was higher than that of HIV-negative patients (27 years).

**TABLE 2:** Description and comparison of socio-demographic and clinical characteristics of human immunodeficiency virus-positive and human immunodeficiency virus-negative patients.

Variable	HIV-positive				HIV-negative				*p*-value
	*n*	%	Median	IQR	*n*	%	Median	IQR	
% of total *n*	40	21.1	-	-	150	78.9	-	-	-
Age (years)	-	-	32	25–36	-	-	27	20–33	0.006*
EPDS score (*n*)	-	-	13	5–18	-	-	11	5–17	0.883
**Significant depressive symptoms: EPDS score > 13**	18	45.0	-	-	64	42.7	-	-	0.858
**Relationship status**	-	-	-	-	-	-	-	-	0.085
*n*	40	-	-	-	147	-	-	-	-
Ongoing relationship % (*n*)	27	67.5	-	-	119	81.0	-	-	-
**Highest level of education**	-	-	-	-	-	-	-	-	0.120
*n*	37	-	-	-	140	-	-	-	-
LSEN school	2	5.4	-	-	6	4.3	-	-	-
Primary school	1	2.7	-	-	9	6.4	-	-	-
High school	12	32.4	-	-	59	42.1	-	-	-
Matric	16	43.2	-	-	50	35.7	-	-	-
Higher education	6	16.2	-	-	16	11.4	-	-	-
**Social support**	-	-	-	-	-	-	-	-	0.042*
*n*	40	-	-	-	148	-	-	-	-
Poor	4	10.0	-	-	7	4.7	-	-	-
Fair	22	55.0	-	-	59	39.9	-	-	-
Good	14	35.0	-	-	82	55.4	-	-	-
**Substance use in pregnancy**	-	-	-	-	-	-	-	-	0.654
*n*	40	-	-	-	150	-	-	-	-
Substance use in pregnancy % (*n*)	9	22.5	-	-	28	18.7	-	-	-
**Unplanned pregnancy**	-	-	-	-	-	-	-	-	0.677
*n*	40	-	-	-	149	-	-	-	-
Unplanned pregnancy % (*n*)	32	80.0	-	-	113	75.8	-	-	-
**Intimate partner violence**	-	-	-	-	-	-	-	-	1.000
*n*	39	-	-	-	147	-	-	-	-
Intimate partner violence % (*n*)	6	15.3	-	-	22	15.0	-	-	-

LSEN, learners with special education needs; HIV, human immunodeficiency virus; EPDS, Edinburgh Postnatal Depression Scale; IQR, interquartile range.

* , Statistical significance.

**FIGURE 2:**
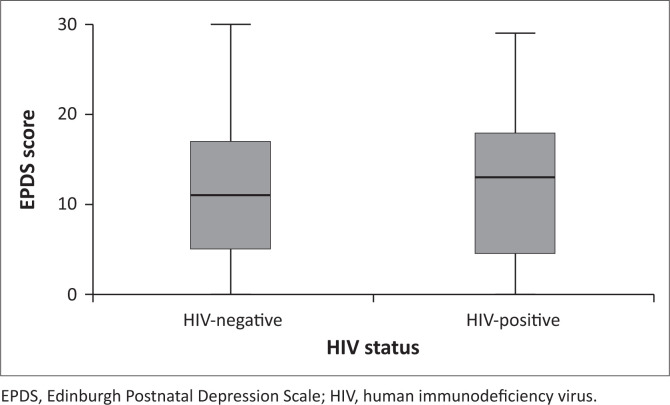
Edinburgh Postnatal Depression Scale scored (median and interquartile range) for human immunodeficiency virus-negative and human immunodeficiency virus-positive patients attending the maternal mental health clinic at Chris Hani Baragwanath Hospital.

The median EPDS score was higher among HIV-positive patients compared to HIV-negative patients (Figure 2). Although a greater proportion of HIV-positive patients (45.0%, *n* = 18) had significant depressive symptoms compared to HIV-negative patients (42.7%, *n* = 64), no statistically significant association was found between HIV status and EPDS score (*p* = 0.883).

A higher proportion of HIV-negative patients (81.0%, *n* = 119) were in an ongoing relationship compared to HIV-positive patients (67.5%, *n* = 27). However, no significant association was found between HIV status and relationship status (*p* = 0.085).

A greater proportion of HIV-positive patients had completed matric or higher education (59.5%, *n* = 22), compared to HIV-negative patients (47.1%, *n* = 66). However, there was no statistically significant relationship between HIV status and educational attainment (*p* = 0.120).

**FIGURE 3:**
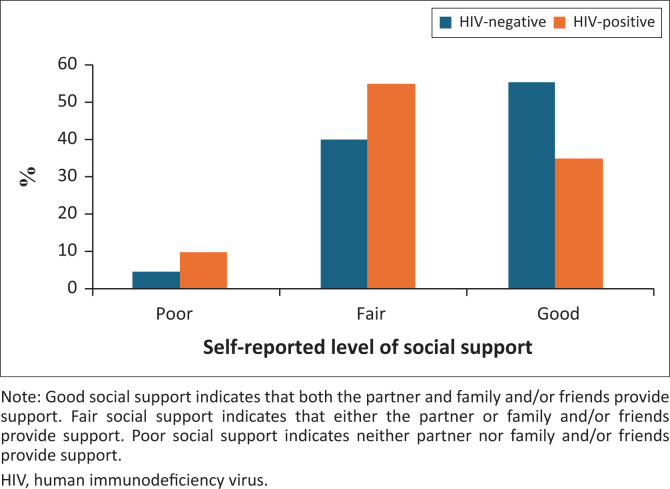
Self-reported level of social support among human immunodeficiency virus-negative and human immunodeficiency virus-positive patients attending the maternal mental health clinic at Chris Hani Baragwanath Hospital.

Poor social support was more prevalent among HIV-positive patients (10.0%, *n* = 4) compared to HIV-negative patients (4.7%, *n* = 7), as shown in Figure 3. A statistically significant association was found between HIV status and level of social support (*p* = 0.042). A multiple linear regression analysis indicated that social support was a significant positive predictor of EPDS score (*p* = 0.017), while HIV status did not have a significant effect (*p* = 0.886) (Table 3).

**TABLE 3:** Multiple linear regression analysis for Edinburgh Postnatal Depression Scale scores.

Independent variables	Beta coefficient	s.e.	*p*-value
Social support	−5.764	2.401	0.017*
HIV status	−0.197	1.375	0.886

HIV, human immunodeficiency virus; s.e., standard error.

* , Statistical significance.

The prevalence of substance use during pregnancy was higher among HIV-positive patients (22.5%, *n* = 9) compared to HIV-negative patients (18.7%, *n* = 28). There was no statistically significant association found between HIV status and substance use in pregnancy (*p* = 0.654).

The rate of unplanned pregnancies was higher amongHIV-positive patients (80.0%, *n* = 32) compared to HIV-negative patients (75.8%, *n* = 113) (Figure 1), with no statistically significant association observed (*p* = 0.677).

The prevalence of intimate partner violence was similar among HIV-positive and HIV-negative patients, with no statistically significant association between HIV status and intimate partner violence (*p* = 1.000).

## Discussion

### Key findings

This study identified high rates of significant depressive symptoms, substance use in pregnancy, unplanned pregnancies, and intimate partner violence among both HIV-positive and HIV-negative women. There was no significant association observed between EPDS scores and HIV status. However, HIV-positive women reported significantly lower levels of social support compared to their HIV-negative counterparts.

### Significant depressive symptoms

The high prevalence of significant depressive symptoms in this study (43.2%, *n* = 82) aligns with findings from a study by Phukuta et al.^19^ who reported a prevalence of 38.8 % (*n* = 88). However, prevalence rates across studies vary greatly. In a study by Mokwena et al.^20^ the prevalence of probable postpartum depression (EPDS score ≥ 13) in Gauteng was reported to be substantially lower (21%, *n* = 50). Several factors could account for differences between prevalence rates. Firstly, variations in sample size may influence prevalence estimates. This study’s sample size was small compared to previous studies,^19,20^ limiting generalisability. Secondly, the maternal mental health clinic at CHBAH is a specialised facility that receives referrals of patients identified as high-risk. This may further explain the observed high rate of significant depressive symptoms compared to studies based in community centres. Additionally, EPDS cut-off scores differ among studies, with some using ≥ 13 and others > 13 to indicate probable depression.^19,20^ Furthermore, unlike previous research that focused exclusively on postpartum women, this study included women across the perinatal period from pregnancy until 6 months postpartum. Rates of perinatal depression may differ from postpartum depression, suggesting that further research into perinatal mental health is warranted. Despite some variability, existing literature consistently indicates that South Africa has amongst the highest prevalence rates of postpartum depression globally.^2^ Contributing factors likely include low socio-economic status, gender-based violence and poor social support.^2^

### Substance use in pregnancy

In this study, 19.5% (*n* = 37) of women reported substance use during pregnancy, indicating a substantial burden of antenatal substance exposure. The prevalence of substance use in pregnancy in this study surpasses that observed in a previous study conducted in KwaZulu-Natal (14.4%, *n* = 32),^21^ yet remains substantially lower than the prevalence of 45% (*n* = 178) previously reported among pregnant women in Gauteng.^22^ Variations in prevalence observed between provinces may reflect differences in urban versus rural substance accessibility, socio-economic conditions and disparities in sample sizes. In addition, prevalence rates may be affected by patient under-reporting due to recall bias or stigma. Although the specific substances used during pregnancy were not assessed in this study, prior research by Sebothoma et al.^22^ reported that concurrent use of alcohol and tobacco was the most prevalent among pregnant women (63%, *n* = 113), whereas cannabis use was comparatively low (2%, *n* = 4). This is particularly concerning given that South Africa has one of the highest reported rates of foetal alcohol syndrome (FAS) globally.^23^ Infants with FAS are at risk for learning difficulties, poor academic performance, and mental health conditions, placing increasing demands on health, education, and support systems.^23^ For mothers, ongoing substance use in pregnancy could indicate ongoing psychosocial adversities such as poverty, gender-based violence and limited social support. The intergenerational impact of prenatal substance exposure underscores the importance of non-judgemental screening during pregnancy for substance use and interventions to address psychosocial risk factors.

### Unplanned pregnancies

The prevalence of unplanned pregnancies in this study (76.7%, *n* = 145) is noted to be higher than that of previous studies conducted in South Africa. In a study based at a primary health care clinic in KwaZulu-Natal, 64.3% (*n* = 211) of women reported having unintended pregnancies.^24^ Additionally, a cross-sectional study by Mokwena et al.^20^ reported a markedly lower prevalence rate of unplanned pregnancies in Gauteng (46%, *n* = 111). The higher observed prevalence rate in this study may be attributed to differences in study populations. Specifically, this study included women with prior mental health conditions or those who developed mental illness during pregnancy. A prior study by Galvin et al.^25^ found that only 44.7% (*n* = 85) of mentally ill women of childbearing age used contraception consistently, suggesting that low rates of contraception use may contribute to the high rate of unplanned pregnancies in this population. Unplanned pregnancies are associated with an increased risk of both antenatal and postnatal depression.^19,20,24,26^ Additionally, pregnancies resulting from contraception failure may further heighten the risk of maternal depressive symptoms,^19^ and increase the likelihood of foetal exposure to teratogenic medications or substances.

### Intimate partner violence

Intimate partner violence remains a major concern in South Africa.^21^ In this study, 15.1% (*n* = 28) of participants reported exposure to intimate partner violence. This rate is similar to that observed in KwaZulu-Natal, where 16.6% (*n* = 37) of women reported physical violence.^21^ In the present study, intimate partner violence was not divided into physical, verbal, or sexual violence, but was grouped into a single category. This methodological approach may account for differences in the prevalence rate compared to other studies. Under-reporting of intimate partner violence may also affect the results. Prior research has established a link between intimate partner violence and antenatal depression.^26,27^ Intimate partner violence is one of many social challenges confronting South African women. Despite multiple initiatives to address social barriers faced by South African women, further progressive action is needed.

### Comparison of human immunodeficiency virus-positive and human immunodeficiency virus-negative patients

In this study, 21.1% (*n* = 40) of participants were HIV-positive, closely aligning with the 2024 national estimate of approximately 20% among women of reproductive-age.^11^ HIV-positive patients were found to have a higher median age compared to HIV-negative patients. This age difference may reflect improvements in HIV prevention strategies, access to healthcare, and educational initiatives among younger women in South Africa, which have been associated with declining HIV incidence in younger reproductive-age populations, although further research is required to confirm this trend.^28^

The prevalence of significant depressive symptoms (EPDS > 13) was marginally higher among HIV-positive women (45.0%, *n* = 18) compared to the HIV-negative women (42.7%, *n* = 64). Human immunodeficiency virus has been shown to be an independent risk factor for depression,^13^ which can account for the slightly higher prevalence observed in the HIV-positive group. There was no statistically significant association found between HIV status and EPDS scores. This finding is consistent with previous research conducted in KwaZulu-Natal comparing EPDS scores among newly diagnosed HIV-positive pregnant women and their HIV-negative counterparts.^5^ It is important to note that small sample sizes in both studies have limited the statistical power to detect a meaningful correlation. Based on a systematic review of 23 studies (including six based in South Africa), HIV-positive women were found to have a significantly higher odds ratio of antenatal and postnatal depressive symptoms compared to HIV-negative women.^13^ Further research using larger sample sizes is required to clarify the relationship between depressive symptoms and HIV status.

A key finding in this study was that HIV-positive women reported significantly lower levels of social support compared to HIV-negative women. This disparity may be partially attributed to the stigma associated with HIV, which can lead to social withdrawal and isolation. Previous research has demonstrated that HIV infection and mental illness are independently associated with stigma and psychological distress.^29^ Moreover, the co-occurrence of these conditions may expose women to compounded forms of stigma, which could contribute to diminished social support among HIV-positive women. Differences in socio-demographic characteristics between the two groups may also account for this disparity. The mean age was higher among HIV-positive women compared to their HIV-negative counterparts. Older women may face greater caregiving responsibilities and have fewer available support networks, which may reduce both actual and perceived social support. Social support has been consistently identified as a protective factor against both antenatal and postnatal depression,^20,27^ while poor support has been significantly associated with elevated risk of postnatal depression.^26,30^ Women who have poor social and financial support have been found to have an increased odds of intimate partner violence and repeated episodes of intimate partner violence.^31^ Consequently, HIV-positive women with poor social support may be particularly vulnerable to worsening depressive symptoms. A related study conducted in KwaZulu-Natal found that pregnancies were more likely to be planned when women were employed and in a stable relationship.^24^ Financial and social stability are key factors in supporting pregnancy planning and maternal well-being.

### Strengths and limitations

This study was conducted at a specialist clinic within a tertiary hospital setting. Given the scarcity of specialised maternal mental health clinics in South Africa, these findings contribute meaningfully to the limited body of research in this field. This study identified a high prevalence of perinatal depressive symptoms among women referred for specialist care, underscoring the significant mental health burden within this population. In addition, it offered a comparative analysis of associated risk factors among HIV-positive and HIV-negative perinatal women.

However, several limitations should be noted. The study’s cross-sectional design limits the ability to draw causal inferences from the associations observed. The sample size was relatively small, which may limit the generalisability of the findings. In addition, as the REDCap database was newly implemented (January 2024) at the CHBAH maternal mental health clinic, several records were incomplete and were subsequently excluded. The use of the EPDS presents another limitation. While the EPDS has demonstrated validity for identifying depressive symptoms up to 6 months postpartum,^32^ current guidelines recommend its use until 4–6 weeks postpartum.^17^ Extending the use of EPDS up to 6 months postpartum may impact its accuracy. Lastly, data on intimate partner violence, substance use during pregnancy, and level of social support were based on self-report, introducing the potential for under-reporting due to stigma, recall or reporter bias.

### Implications and recommendations

The findings of this study highlight perinatal depressive symptoms as a significant public health concern in South Africa, particularly among HIV-positive women. Human immunodeficiency virus -positive women appear to be at a heightened risk, partly due to limited social support, which may exacerbate depressive symptoms. Further research with a larger sample size is recommended to strengthen the evidence base and direct policy and clinical practice. Additionally, longitudinal studies assessing depressive symptoms across different stages of the perinatal period may enhance early identification and timely intervention of women in need of mental health care.

## Conclusion

This study found high rates of significant depressive symptoms among perinatal women. The complex social, economic, and medical challenges prevalent in South Africa likely contribute to this ongoing burden. These findings indicate an urgent need to improve maternal health services in South Africa. Perinatal depression poses serious risks to both mother and child, underscoring the urgent need for targeted interventions. High rates of unplanned pregnancies and intimate partner violence were observed. Human immunodeficiency virus -positive women were significantly more likely to report poor social support. Poor social support, a recognised risk factor for perinatal depression, highlights the critical need to address stigma and enhance community support systems for women within the perinatal period.
